# Evaluation of low-intensity pulsed ultrasound on doxorubicin delivery in 2D and 3D cancer cell cultures

**DOI:** 10.1038/s41598-020-73204-y

**Published:** 2020-09-30

**Authors:** Miglė Paškevičiūtė, Indrė Januškevičienė, Kristina Sakalauskienė, Renaldas Raišutis, Vilma Petrikaitė

**Affiliations:** 1 Laboratory of Drug Targets Histopathology, Institute of Cardiology, Lithuanian University of Health Sciences, Sukilėlių pr. 13, 50162 Kaunas, Lithuania; 2 Ultrasound Research Institute, Kaunas University of Technology, K. Baršausko g. 59, 51423 Kaunas, Lithuania; 3 Department of Electrical Power Systems, Faculty of Electrical and Electronics Engineering, Kaunas University of Technology, Studentu g. 50, 51368 Kaunas, Lithuania; 4 Institute of Physiology and Pharmacology, Faculty of Medicine, Lithuanian University of Health Sciences, A. Mickevičiaus g. 9, 44307 Kaunas, Lithuania

**Keywords:** Experimental models of disease, Translational research, Cancer models

## Abstract

The aim of our study was to evaluate the influence of low-intensity pulsed US on the delivery of doxorubicin (DOX) into MDA-MB-231 triple-negative breast cancer and A549 non-small cell lung cancer cell 2D and 3D cultures. US with pulse repetition frequency of 10 Hz and 1 MHz center frequency was generated with peak negative pressure of 0.5 MPa and 50% duty cycle. SonoVue microbubbles were used. Spheroids were formed using 3D Bioprinting method. DOX delivery in 2D and 3D cultures was assessed using fluorescence microscopy. US without the addition of microbubbles did not enhance the penetration of DOX into monolayer-cultured cells and tumor spheroids. In the presence of microbubbles US improved the delivery of DOX into the edge end middle zones of A549 and MDA-MB-231 spheroids. Application of low-intensity pulsed US in combination with microbubbles may be a promising approach to enhance the delivery of DOX into tumor spheroids.

## Introduction

Poor drug delivery into cancer cells is one of the biggest concerns in anticancer therapy. It is the result of various factors such as poor tumor vascularization, hypoxia, or increased interstitial fluid pressure in tumors^1,2^. Insufficient drug concentration in the tumor limits the therapeutic efficacy of anticancer agents. Therefore, it is very important to find novel strategies that would enhance anticancer drug transport into cancer cells thus increasing their efficacy. One of the methods that recently are gaining more and more attention from scientists is sonoporation caused by ultrasound (US).

The first clinical application of US was recorded in the middle of the twentieth century for the imaging of tissues and blood flow^3^. Nowadays it became one of the most common diagnostic imaging techniques^4^. US imaging is safe, painless, non-invasive, non-ionizing, relatively inexpensive, and simple. US can also be focused on the target tissues and organs, thus improving the selectivity of affected areas and reducing the side effects in healthy tissues^5^. Due to these advantageous characteristics, over the last decades, US also has gained a growing interest in the field of drug delivery. There are two main mechanisms by which US may affect drug transport into cells. In the presence of US, the fluid starts moving in oscillatory motions. It was observed that these motions of the fluid may enhance the diffusion of the molecules through the cell membrane^6^.

Another mechanism includes the oscillation of microscopic (1–4 μm in diameter) gas bubbles. In order to enhance US contrast between blood and surrounding tissues, special inert gas microbubbles coated with a shell made of lipids, polymers, and proteins are used during US imaging procedure. According to research, US produces expansion and contraction of microbubbles^7^. This phenomenon is called cavitation. It was observed that cavitation causes the flow of the fluid around the microbubble. Produced shear forces may create small pores in cell membranes^6^. When the intensity of US increases and the acoustic pressure exceeds a certain threshold, the changes of the microbubble volume start being controlled by the inertia of the surrounding liquid^8^. This type of cavitation is called inertial cavitation. During inertial cavitation microbubbles may expand more than twice their radius and collapse, thus producing a shock wave of the surrounding fluid^8^. It is hypothesized that this shock wave can rupture the cell membrane^9^. The collapse of microbubbles may also cause the formation of microjets that can form pores in the cell membrane^10^. Through those ruptures, drugs penetrate into cells via passive diffusion.

Adjustment of ultrasound frequency is important in order to achieve deeper tissues and achieve the valuable effect of drug delivery. US induced formation of pores in cell membranes is called sonoporation. It was estimated that the pore size may vary from 1 nm to several micrometers^11^. Pore resealing occurs very fast and it may take from a few up to 120 s^12^. On the other hand, high intensity focused US may cause an irreversible sonoporation^13^. This leads to permanent pore formation in membranes, lysis, and eventually to cell death and tissue damage. US frequency is a key parameter for the depth of US penetration. High frequency US has a short (small) depth of penetration, therefore to achieve deeper penetration, low frequency US is used^14^. In order to investigate the effect of US on drug delivery the application of 1 MHz and a low pulse repetition frequency US is recommended^15^. Pulsed US instead of a continuous single tone burst US was used to create the flow of fluid but to prevent the irreversible damage of cells and cell death^16,17^.

The aim of our study was to evaluate the influence of microbubble-assisted low-intensity pulsed US on the delivery of doxorubicin (DOX) into monolayer-cultured A549 non-small cell lung cancer and MDA-MB-231 triple-negative breast cancer cells (2D cultures) and tumor spheroids (3D cultures). 3D cultures of cells is a spatially arranged group of cells and they are not limited by a single plane (monolayer) only, mimicking the real spatial arrangements of cells within a tissue of the real tumour. Thus, tumor spheroids provide a better representation of the real tumors and their spatial complexity, and the experiments in 3D models provide more accurate data than the studies with typical 2D cell cultures^18^.

## Results

### Effect of US on cytotoxicity of DOX

Neither the microbubbles nor US separately nor their combination had any effect on the viability of cells. The use of microbubbles without US and the application of US without microbubbles did not increase the cytotoxicity of DOX. A statistically significant difference compared to the control group was found only when the combination of DOX, microbubbles and US was used. In this case, the cytotoxicity of DOX was increased by approximately 5.7% (Fig. S1 in the Supplementary Information).

### Effect of US on DOX delivery in cancer cells (2D cultures)

**Figure 1:**
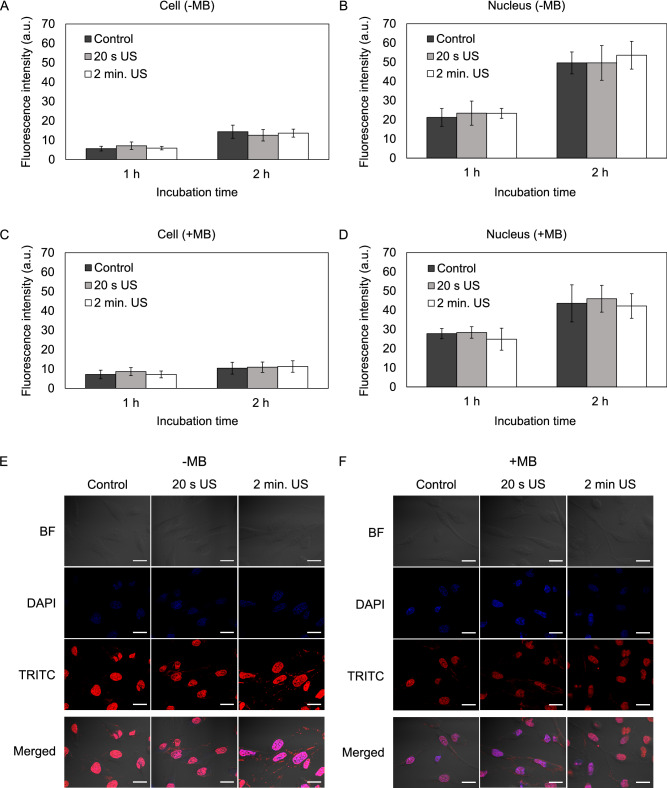
The effect of ultrasound (US) and microbubbles (MB) on doxorubicin (DOX) delivery into monolayer-cultured MDA-MB-231 cells. (**A**) DOX fluorescence intensity in cells affected with US without the addition of MB. (**B**) DOX fluorescence intensity in cell nucleus after the application of US without the addition of MB. (**C**) DOX fluorescence intensity in cells affected with US and MB. (**D**) DOX fluorescence intensity in MDA-MB-231 cell nucleus after the application of US with MB. (**E**) Images of cells after 2 h incubation with DOX. Magnification 600 ×. Scale bar = 50 μm. *US* ultrasound, *MB* microbubbles.

Neither applied separately nor in combination with microbubbles US did enhance DOX delivery in monolayer-cultured cancer cells and nucleus (Fig. 1A–F). The fluorescence intensity of DOX in both cell lines affected by US for 20 s or 2 min of US did not differ from the control group after 1 h and 2 h of incubation. The addition of the microbubbles did not increase the fluorescence intensity of DOX into cells and nucleus as well.

### Effect of US on DOX delivery in spheroids (3D cultures)

**Figure 2:**
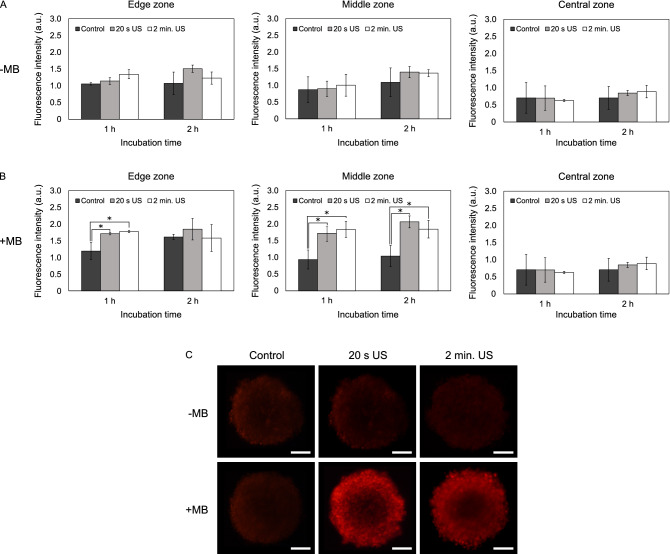
The effect of ultrasound (US) and microbubbles (MB) on doxorubicin (DOX) delivery into A549 spheroids. (**A**) DOX fluorescence intensity in different spheroid areas after the application of US without the addition of MB. (**B**) DOX fluorescence intensity in different spheroid areas after the application of US with the addition of MB. (**C**) Images of spheroids after 2 h incubation with DOX. Magnification 100 ×. Scale bar = 100 μm. The asterisks (*) indicate *p* < 0.05. *US* ultrasound, *MB* microbubbles.

**Figure 3:**
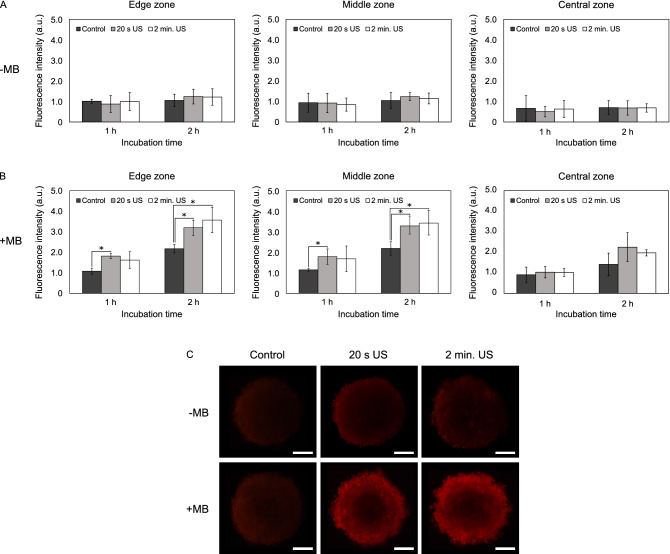
The effect of ultrasound (US) and microbubbles (MB) on doxorubicin (DOX) delivery into MDA-MB-231 spheroids. (**A**) DOX fluorescence intensity in different spheroid areas after the application of US without the addition of MB. (**B**) DOX fluorescence intensity in different spheroid areas after the application of US with the addition of MB. (C) Images of spheroids after 2 h incubation with DOX. Magnification 100 ×. Scale bar = 100 μm. The asterisks (*) indicate *p* < 0.05.

The results were different in 3D models. US without microbubbles did not enhance DOX delivery into A549 and MDA-MB-213 spheroids (Figs. 2A, 3A). When combined with microbubbles US enhanced the penetration of DOX into edge and middle zones of A549 spheroids (Fig. 2B,C). 20 s of US exposure increased the fluorescence intensity into the edge and middle zones of A549 spheroids approximately 1.4- and 1.8-fold after 1 h incubation. After 2 h, incubation DOX delivery increased only in the middle zone (approximately twofold). A significant increase in the fluorescence intensity in the central zone was not observed.

The effect of 2 min of US exposure on DOX delivery into spheroids was similar. The fluorescence intensity into the edge zone increased 1.5-fold and into the middle zone—2.0-fold after 1 h incubation. After 2 h incubation, the amount of DOX in the middle zone increased 1.8-fold.

In MDA-MB-231 spheroids 20 s of US exposure enhanced the delivery of DOX into edge and middle zones both after 1 h and 2 h incubation (Fig. 3B,C). After 1 h the fluorescence intensity in the edge and middle zones increased 1.7- and 1.6-fold, respectively. After 2 h incubation, DOX penetration into both edge and middle zones increased 1.5-fold. No increase in the fluorescence intensity in the central zone was observed.

2 min of US exposure did not enhance DOX delivery into the edge and middle zones of spheroids after 1 h incubation. However, 2 min of US exposure increased the fluorescence intensity into the edge and middle zones 1.6-fold after 2 h incubation.

## Discussion

In some studies, the influence of US was investigated without the addition of microbubbles but US alone was effective in increasing cell membrane permeability to compounds. Forbes et al.^19^ showed that US enhanced the cell permeability only in the presence of microbubbles, meanwhile US alone did not stimulate the intracellular uptake of FITC-dextran. Our data is consistent with these findings since we also did not observe any positive effect on the delivery of DOX into cells and spheroids without the addition of microbubbles. This could mean that microbubbles are crucial to enhance drug uptake into cell cultures.

Even though during the experiments with monolayer-cultured cells we tried to follow the descriptions of the methods applied by other laboratories, our data contradict some results obtained by other scientists. We observed that microbubble-assisted US did not enhance the delivery of DOX into monolayer-cultured cells. However, some studies demonstrated that US combined with microbubbles may enhance the delivery and therapeutic effect of anticancer agents. For example, Van Wamel et al.^20^ showed that US in combination with BR14 microbubbles enhanced the permeability of bovine endothelial cells using propidium iodide as a probe. Such a difference between our findings and the results shown by other scientists may depend on the type of microbubbles used in studies. Escoffre et al.^21^ showed that the combination of US and Vevo MicroMarker microbubbles slightly enhanced DOX uptake into monolayer cultured MDA-MB-231 cells and increased DOX-induced cell death, meanwhile SonoVue microbubbles did not enhance the effect of DOX on these cells. This means that not all types of microbubbles exert the same effect on the pore formation in the cell membrane.

Nevertheless, we found out that SonoVue microbubbles are not efficient for US-assisted drug delivery in monolayer-cultured cells, certain studies showed that these microbubbles combined with US may also enhance drug uptake into 2D cell cultures. Lamanauskas et al.^22^ demonstrated that US combined with SonoVue microbubbles increased bleomycin-induced U-87 and HCT-116 cell death by 30%. The authors hypothesize that this effect was determined by the enhanced uptake of bleomycin. Such disagreement between the findings from this study and our data might be determined by the tendency of the microbubbles to move upwards to the surface of the medium. In this way, the number of microbubbles closely surrounding the cells decreases, thus diminishing the possible effect or creating the variability between the results. In order to prevent this problem, it would be worthy to think of some modifications that would hinder the microbubbles from floating on the surface of the medium.

Even though US with microbubbles was not effective in 2D cell cultures, we showed that it did enhance the penetration of DOX into tumor spheroids. To our knowledge, there are no published data of the application of US in combination with SonoVue microbubbles on drug delivery to tumor spheroids. However, other studies demonstrated the positive results of other types of microbubbles in combination with US. Grainger et al.^16^ showed that pulsed US exposure in combination with Optison microbubbles enhanced the penetration of fluorescent nanoparticles (20–100 nm) into the core zone of MCF-7 breast cancer spheroids 3–20-fold. Leenhardt et al.^23^ estimated that the application of US and microbubbles in combination with gemcitabine decreased the viability of pancreatic cancer spheroids, compared to the group treated only with gemcitabine.

According to our knowledge, for now, there exists one clinical trial evaluating the use of low-intensity pulsed US and microbubbles to treat cancer^24^. In this phase I study US and SonoVue microbubbles were combined with gemcitabine to treat inoperable pancreatic cancer. The data showed the sonoporation combined with gemcitabine significantly increased the survival of patients compared to a historical cohort of patients (control group). There was no additional toxicity observed. However, the study was small (n = 10) and larger scale studies are needed.

## Conclusions

Low-intensity pulsed US separately and in combination with microbubbles does not enhance the delivery of DOX into monolayer-cultured A549 and MDA-MB-231 cells. US without microbubbles also does not improve the delivery of DOX into tumor spheroids. However, microbubble-assisted US increases the amount of DOX into the edge and middle zones of spheroids. Application of low-intensity pulsed US in combination with microbubbles may be a promising approach to enhance the delivery of DOX into tumor spheroids.

## Material and methods

### Materials

DOX hydrochloride was purchased from Abcam (Cambridge, UK). Microbubbles SonoVue (MB) were bought from Bracco (Milan, Italy).

### Cell cultures

The non-small cell lung cancer cell line A549 and the triple-negative breast cancer line MDA-MB-231 were purchased from the American Type Culture Collection (ATCC, Manassas, VA, USA). Both cell lines were cultured in Dulbecco's Modified Eagle Medium that contained 10,000 U/mL penicillin, 10 mg/mL streptomycin, and 10% fetal bovine serum. Media and all the supplements were bought from Gibco (Carlsbad, CA, USA). Cells were incubated in a humidified atmosphere with 5% CO_2_ at 37 °C.

### US parameters

Ultrasonic transducer V303 with diameter of 12.7 mm, working at 1 MHz centre frequency (Olympus Corp., Japan), was used for the sonication of the 2D and 3D cancer cell cultures. The sinusoidal pulsed excitation signal with 10 Hz pulse repetition frequency and amplitude of 0.8 V (peak to peak) was generated (generator 81150A, Agilent Technologies, Indianapolis, IN) and amplified by developed in a lab external radio frequency amplifier of 40 dB (peak to peak amplitude of 80 V) in order to reach the acoustic peak negative pressure amplitude of 0.5 MPa, the duty cycle was set at 50% during sonication. The output pressure of the transducer was calibrated with Onda immersion measurement system and HGL-0400 capsule hydrophone (“Onda Corporation”, Sunnyvale ,USA). We have selected to study 0.5 MPa at 1 MHz according to the recommendations provided by Wood et al.^25^.

### Effect of US on cytotoxicity of DOX

Cell viability was assessed by 3-(4,5-dimethylthiazol-2-yl)-2,5-diphenyltetrazolium bromide (MTT) assay. The prepared suspension of the cells was poured in a plastic cuvette (height 45 mm, width 10 mm) in a volume of 1.5 ml (the total number of cells in the cuvette was 5 × 10^5^). The cuvette was immersed in a deionized water bath and sonicated for 20 s. DOX concentration of 10 µM was used with or without 8.3 µL of microbubbles.

The suspension was centrifuged at 1000 rpm for 4 min. The supernatant was replaced with fresh medium. The suspension was poured into 96-well plate in a volume of 100 µL/well, so that each well contained 5000 cells. After 72 h 10 µL MTT solution (5 mg/mL) was added to the wells and incubated for 4 h in a humidified atmosphere with 5% CO_2_ at 37 °C. The medium was removed from the plates and the formazan crystals were dissolved in 100 μL of DMSO. The number of viable cells was assessed spectrophotometrically by measuring the absorbance of the solutions at wavelengths of 570 nm and 630 nm. The data were analyzed using Microsoft Office Excel 2016 (Microsoft Corporation, Redmond, WA, USA).

### Drug delivery in monolayer-cultured cells

The cells were seeded in 24-well plates on collagen-coated coverslips in a volume of 500 μL (50,000 cells/well) and incubated for 48 h in a humidified atmosphere with 5% CO_2_ at 37 °C. Later, the medium was replaced by the fresh medium that contained 10 µM DOX. The plate was positioned above the US transducer and the cells were affected by US for 20 s or 2 min. The Aqualene elastomer couplant (Olympus Corp., Japan) possessing solid-state and thickness of 25 mm was used as the intermediate low attenuating material between the radiating surface of ultrasonic transducer and bottom of the plate in order to ensure far field fixed distance and avoid variations of acoustic pressure. For acoustic contact at both boundaries of elastomer, the coupling gel was used. The visible graphical contours (circularly shaped with cross in the middle) were depicted by permanent black colour marker on both front and back surfaces of the elastomer couplant. It was performed in order to ensure the repeatable positioning of it over the ultrasonic transducer, also positioning of the particular well at the centre of ultrasonic beam being transmitted by ultrasonic transducer through the mentioned elastomer couplant. After 1 or 2 h of incubation, the medium was removed, the cells were washed with PBS, fixed with 4% paraformaldehyde (Thermo Scientific, Waltham, MA, USA) solution in PBS and stained with 4′,6-diamidino-2-phenylindole (DAPI; Thermo Scientific). The photos were taken using fluorescence microscopy (Olympus IX73) and the penetration of DOX into whole cells and their nucleus was evaluated using *ImageJ* software (National Institutes of Health).

### Drug delivery in tumor spheroids

The spheroids were formed using 3D bioprinting method following the method described by Tseng et al.^26^ Each spheroid consisted of 2000 cancer cells (A549 or MDA-MB-231) and 2000 human fibroblasts. The spheroids were transferred into 24-well plates and 200 µL of fresh medium that contained 10 μM DOX with or without 20 µL MB (prepared in DMEM) was added to each well. The plate was positioned above the US transducer and affected by US for 20 s or 2 min. The same Aqualene elastomer couplant of thickness 25 mm was used as the intermediate low attenuating material between the radiating surface of the ultrasonic transducer and the bottom of the plate. For acoustic contact at both boundaries of elastomer, the coupling gel was used as well. After that, the spheroids were washed with PBS, fixed with 4% paraformaldehyde solution. The penetration of DOX into spheroids was assessed using fluorescence microscopy (Olympus FLUOVIEW FV1000) and ImageJ software by evaluating fluorescence intensity every single degree from the spheroid center to the edge around the whole spheroid. During the analysis, the spheroids were relatively divided into three zones: edge zone (0–50 µM), middle zone (100–150 µM), and central zone (200–225 µM).

### Statistical analysis

Statistical analysis was done using Microsoft Office Excel 2016. Each experiment was repeated at least three times independently and the results were presented as mean ± standard deviation. The analysis was performed using Student’s *t* test, and *p* values were calculated. A value of *p* < 0.05 was considered a significant difference.

### Ethical approval

This article does not contain any studies with human participants or animals performed by any of the authors.

### Informed consent

For this type of study, formal consent is not required.

## Supplementary information

Supplementary Figure S1.
