# Supplementary material for: Evaluation of low-intensity pulsed ultrasound on doxorubicin delivery in 2D and 3D cancer cell cultures
Source: Sci Rep. 2020 Sep 30;10:16161. doi: 10.1038/s41598-020-73204-y (PMC7527335; doi:10.1038/s41598-020-73204-y)
Supplement: Supplementary file 1 — Supplementary Figure S1. [file 41598_2020_73204_MOESM1_ESM.docx]

**Evaluation of low-intensity pulsed ultrasound on doxorubicin delivery in 2D and 3D cancer cell cultures**

Miglė Paškevičiūtė^1^, Indrė Januškevičienė^1^, Kristina Sakalauskienė^2^, Renaldas Raišutis^3^, Vilma Petrikaitė^1,4^

^1^Laboratory of Drug Targets Histopathology, Institute of Cardiology, Lithuanian University of Health Sciences, Sukilėlių pr. 13, LT-50162, Kaunas, Lithuania

^2^Ultrasound Research Institute, Kaunas University of Technology, K.Baršausko g. 59, LT-51423 Kaunas, Lithuania

^3^ Department of Electrical Power Systems, Faculty of Electrical and Electronics Engineering, Kaunas University of Technology, Studentu g. 50, LT-51368 Kaunas, Lithuania

^4^Institute of Physiology and Pharmacology, Faculty of Medicine, Lithuanian University of Health Sciences, A. Mickevičiaus g. 9, LT-44307, Kaunas, Lithuania

Supplementary Figure S1


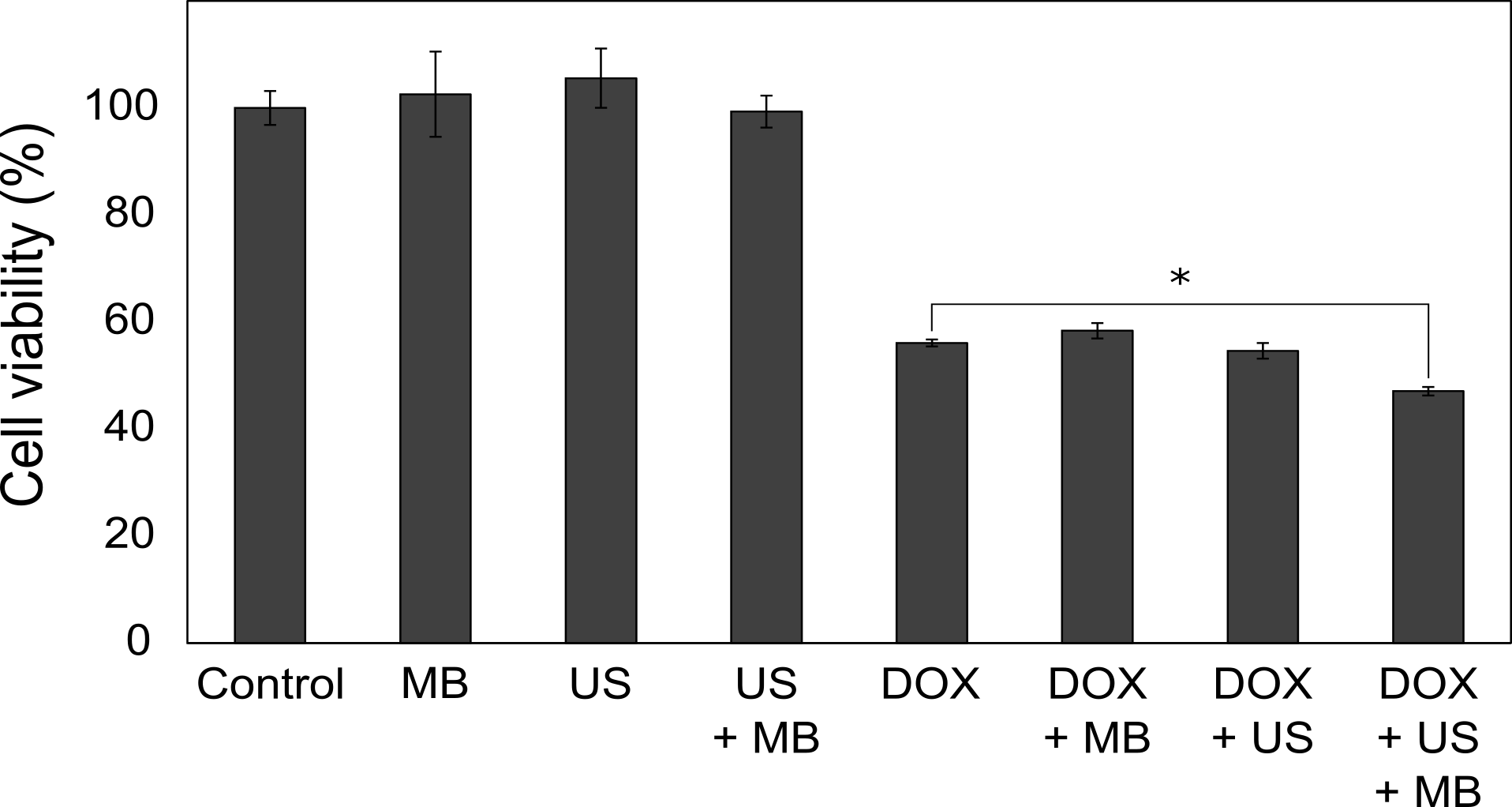


**Supplementary Figure S1** The viability of A549 cells after 72 h of incubation.

Abbreviations: Dox, doxorubicin, US, ultrasound; MB, microbubbles. The asterisks (*) indicate p < 0.05.
